# Effect of Pubertal Suppression on Linear Growth and Body Mass Index; a Two-Year Follow-Up in Girls with Genetic Short Stature and Rapidly Progressive Puberty

**Published:** 2014-06

**Authors:** Zohreh Karamizadeh, Anis Amirhakimi, Gholamhossein Amirhakimi

**Affiliations:** Department of Pediatrics, Shiraz University of Medical Sciences, Shiraz, Iran

**Keywords:** Short Stature; Puberty; Gonadotropin Releasing Hormone Agonists; Body Mass Index

## Abstract

***Objective:*** Gonadotropin-Releasing Hormone agonists (GnRHa) are used to improve the final adult height in short stature children. There are limited studies which address the potential side effect of these agents: excessive weight gain. We have followed girls with rapidly progressive puberty receiving GnRHa and results were focused on the effect of treatment on final height, weight and body mass index

***Methods:*** Thirty girls between 8.5 and 12 years with short stature and predicted adult height of less than 155 cm were enrolled in the study. All had rapidly progressive puberty. Weight and height measurements were done at the beginning of treatment, 6 and 12 months after starting and 6 and 12 months after the cessation of treatment. Bone age and stages of puberty were estimated at the beginning of treatment, after 12 months of starting and 12 months after the treatment was stopped.

***Findings*** ***:*** Predicted adult height (PAH) changes during treatment were not significant. There was no significant difference between final height and weight according to the body mass index (BMI), PAH or bone age.

***Conclusion:*** We conclude that girls with genetic short stature and rapidly progressive puberty will not benefit receiving a one-year course of GnRHa and there is no significant difference between the final height and final weigh among children according to BMI.

## Introduction

Short stature has always been a serious mind-occupying concern of parents all over the world and different therapeutic approaches are now being used by pediatric endocrinologists^[^^1^^-^^5^^]^. Almost 20% of the adult height is achieved during the pubertal growth^[^^6^^-^^12^^]^and this might be the reason most parents seek medical help before or in the early stages of puberty of their children in order to do something that can help them achieve a taller stature. Studies have been performed to compare different methods and controversial results are available, but the most prominent feature of all studies, is the need for further evaluation^[^^1^^-^^5^^]^.

 Gonadotropin-Releasing Hormone agonists (GnRHa) are widely used to desensitize the pituitary axis for secreting endogenous GnRH and suppress the progression of puberty^[^^13^^-^^18^^]^. As a result, these agents postpone bone maturation and reduce the rate of epiphysiseal maturation due to the lack of steroid sex hormones and help improve the final adult height. Despite extensive research, the best time to start and end the treatment with GnRHa and the positive effect they have on the final height are still not clear.

 An important point is the concordance between the clinical pubertal development and the growth spurt^[^^4^^]^. Growth acceleration in girls, generally takes place prior to or during the first year of breast development, the pattern of which has large individual variations. Considering Tanner staging, 40% of girls have their peak growth velocity at breast stage 2 (B2), 30% in B3, 20% in B4 and 10% before any breast development occurs (B1).

 The amount of body fat is one other important component of adolescent growth during puberty. It is well known that puberty and growth both are accelerated with common obesity^[^^1^^]^.

 It is suggested that excessive weight gain might be an unfavorable side effect of the treatment with GnRHa and there are limited studies addressing this issue^[^^4^^,^^19^^-^^22^^]^.

 In the present study, we have followed girls with rapidly progressive puberty who received GnRH afor pubertal suppression and results were reviewed focusing on the effect of treatment on their final height, weight and body mass index.

## Subjects and Methods

We prospectively followed thirty girls aged between 8.5 and 12 years who referred to the endocrinology clinic due to short stature and had predicted adult height of less than 155 cm. All of our subjects had rapidly progressive puberty (started after the age of 8 yrs) documented by follow-up physical examinations performed in a three-month period before starting any treatments. Subjects enrolled in this study had increasing Tanner’s stage of puberty by at least one point or had presented an additional sign of pebertal progression (e.g. pubic or axillary hair).

*Exclusion criteria:* any additional condition affecting body mass index (BMI) or puberty onset like deficiency of growth hormone, hypothyroidism or congenital adrenal hyperplasia. 

 Treatment with GnRHa (diphereline) was started for all subjects in a dose of 80 mcg/kg every 28 days and continued for 12 months. Weight and height measurements using standard scales, were done at the beginning of treatment, 6 and 12 months after starting the treatment and also 6 and 12 months after the cessation of treatment. Achievement of final height (FH) was defined when the growth rate reached to less than 0.5 cm/year, bone age was more than 15 yrs and bone x-rays showed closed epiphyseal growth plates.

 Bone age was assessed according to the left hand x-ray and was estimated for all subjects at the beginning of GnRHa treatment, after 12 months of starting the treatment and 12 months after the treatment was stopped. Stages of puberty were estimated by expert pediatric endocrinologists using the Tanner staging method at the beginning of treatment, 12 months after the start and 12 months after the cessation of treatment. Bayley-Pinneau method was used for calculation of the predicted adult height (PAH). Target height was measured for all subjects and all of the PAHs were less than the target heights.

 All data were analyzed using SPSS software version 17. Statistical analyses were performed by Repeated Measurement Test, Student t-Test and Pairwise Comparison (Boneferroni Method). Mann-Whitney Test was also used for comparing data between different groups. *P* value of less than 0.05 was considered significant for all tests.

 Our study was prepared according to the ethical principles of the Helsinki II declaration. The ethics committee in the Department of Medical Ethics, located in Shiraz University of Medical Sciences, approved the study protocol. Written informed consent was provided by all children and their parents.

***Findings***

Thirty girls aged between 8.5 and 12 years were evaluated and enrolled in the study. All of these girls had their early stages of puberty (breast enlargement) after the age of 8 y (no one had precocious puberty) and all had rapidly progressive puberty confirmed by serial physical examinations during the 3 months before starting the treatment. Patient characteristics are summarized in Table 1. Sexual maturity rate 

**Table 1:** Patient characteristics before starting the treatment

**Parameter**		**Mean (SD)**	**Range**
**Age (y)**		10.5 (1)	8.5-12
**Weight (kg)**		32.56 (0.74)	24-40
**Height (cm)**		137 (0.78)	127-144
**BMI (kg/m** ^2^ **)**		17.5 (1.9)	14.7-21.6
**BMI Percentile for Age (%) **		50.9 (8.5)	10-95
**Bone Age (y)**		11.6 (0.01 )	10-13
**PAH (cm)**		150.9±2.2	141-155
**SMR Breast (%)**	II	10	
	III	53.3	
	IV	36.7	
**SMR Pubic Hair (%)**	I	3.3	
	II	33.3	
	III	36.7	
	IV	26.7	

BMI: Body Mass Index, PAH: Predicted Adult Height, SMR: Sexual Maturity Rating

according to Tanner method was estimated for all subjects in each visit and the details shown in Table 1 correspond to the physical examination performed just before starting the treatment. In 76.7% of girls menarche had not occurred but 23.3% had at least one menstrual cycle before treatment.

 Height was measured 5 times and the increase in height during treatment was statistically significant (*P*<0.001) and was evaluated by subtracting the first height measured from all other measurements (Table 2). The rate of Ht increment during treatment was calculated during 4 periods: first 6 m and second 6 m after beginning the treatment, first 6 m and second 6 m after cessation of treatment. Interestingly, the mean of Ht increment was highest during the first 6 m after beginning of treatment (2.9±0.15 cm/6 m) compared to the other three periods which follow respectively: 2.2±0.12, 2.1±0.14 and 2.2±0.18 (*P*<0.001). Weight was also measured 5 times and the increment was calculated (Table-2) which also showed statistically significant rise during our treatment. BMI was also significantly increased during treatment (*P*<0.001).

 Bone Age assessment is summarized in Table 2. 

Statistical analysis showed that the mean bone age during treatment had a significant increment (*P*<0.001). The mean change in bone age was 1.7±0.5 with a minimum increase of 0.5 and a maximum of 3 years.

 PAH changes during treatment were not significant and the mean PAH one year after treatment cessation was 152 cm (min 144 and max 161). The mean difference in PAH was 1.49±3.74 with a maximum increase of 7.1 cm.

 Pubertal progression ceased after starting treatment in all of our subjects and the Tanner’s staging advanced no more whilst the subjects received the GnRHa.

 The average interval between the cessation of the 1 year treatment and menarche in our patients was 14±7.5 months (min 4 and max 28 months). Final height (FH) was measured for all subjects and had an average of 150.2±3.6 (min 144 and max 157 cm). Average of the Final weight (FW) was 42.7±5 (min 35 and max 53 kg). For better understanding of the effect of the treatment and for possibility of comparing among subjects, we defined three groups according to the BMI, PAH, and bone age as follows:

**Table 2:** Height, Weight and Bone Age during and after treatment

**Measurements**	**Height**		**Weight**		**Bone age**	
	**Mean**	**SE**	**Mean **	**SE**	**Mean **	**SE**
**Before starting treatment**	137.15	0.78	32.57	0.74	11.60	0.05
**6 m after starting treatment**	140.05	0.77	34.49	0.81	--	--
**12 m after starting treatment**	142.25	0.78	36.59	0.84	12.48	0.08
**6 m after cessation of treatment**	144.36	0.76	38.18	0.86	--	--
**12 m after cessation of treatment**	146.58	0.77	40.16	0.91	13.31	0.05

SE: Standard Error

**Table 3:** Comparison of Final Height and Final Weight among different groups

**Groups**	**Final height (cm)**		**Final weight (kg)**	
	**Mean (SD)**	***P.*** ** value**	**Mean (SD)**	***P.*** ** value**
**BMI-1**	149.94 (3.49)	0.5	39.95 (3.74)	0.06
**BMI-2**	150.66 (4.1)		47.68 (2.68)	
**PAH-1**	147.88 (2.5)	0.1	42.46 (5.53)	0.8
**PAH-2**	151.75 (3.52)		43.00 (4.86)	
**Bone age-1**	150.69 (3.84)	0.2	43.73 (5.07)	0.4
**Bone age-2**	148.25 (1.97)		39.00 (2.91)	

SD: Standard deviation; BMI: Body mass index; PAH: Predicted adult height

*BMI Group:* BMI before starting the treatment of below 18 kg/m^2 ^which included 19 of our subjects (63.3%) and the BMI of 18 and above which included 11 subjects (36.7%). Also the BMI one year after the cessation of treatment of below 18 kg/m^2^ of 13 subjects (43.3%) and the BMI of 18 and above that included 17 (56.7%).

*PAH Group:* PAH before starting treatment of less than 150 cm which included 12 of our patients (40%) and the PAH of 150 and above which included 18 patients (60%).

*Bone age Group:* Group 1 were subjects in whom the bone age before starting the treatment was estimated within 1 year of their chronological age and group 2 were those whose bone age was more advanced and had more than 1 year difference with their chronological age. Group 1 included 24 patients (80%) and group 2 consisted only of 6 patients (20 %).

 We compared the final height and final weight in these three groups and we concluded that there is no significant difference between these two parameters among different groups. Data are summarized in Table 3.

 BMI calculated before the start of treatment was compared with the BMI one year after the cessation of treatment and 22 (73.3%) of our patients had no change in BMI, in one (3.3%) patient BMI had decreased and in the other 7 (23.3%) BMI had increased. The mean change of BMI was 1.39 kg/m^2^ ±1.2 (with the most decrease of 0.7 and the maximum increase of 5.18). The reason for the increased BMI is still unclear and requires further investigation. Nevertheless, increased appetite, low physical activity and baseline increased BMI can be predisposing factors.

 No correlation was found between BMI and start of menarche after cessation of treatment. Despite the changes in the BMI, we found no correlation between the difference of BMI and the start of menarche. This correlation was checked in both the BMI before starting the treatment and the BMI one year after its cessation and also compared when BMI was classified into two groups of below and above 18 kg/m^2^ among which the difference was not significant (Table 4).

 We found no significant correlation between the PAH before and after treatment with BMI (*P.* value of 0.07 and 0.9 respectively).

## Discussion

We conclude that girls with genetic short stature and rapidly progressive puberty with relatively early onset, posing them at risk of not attaining their desired adult height, will not benefit receiving a course of one-year treatment with GnRHa.

**Table 4:** Mean duration of menarche after cessation of treatment compared between different groups of Body Mass Index

**Body mass index**		**Mean duration of menarche ( mo)**	***P.*** ** value**
**Before treatment**	< 18 kg/m^2^	14.37	0. 7
	≥ 18 kg/m^2^	13.45	
**After treatment**	< 18 kg/m^2^	15	0.8
	≥ 18 kg/m^2^	13.29	

It is also concluded that BMI can increase significantly but there is no significant difference between the final height and final weight among children with lower or higher BMIs. It means that no advantage exists for girls with lower BMI in gaining taller stature or no disadvantage for obese girls in remaining short despite treatment.

 The literature is limited on the final effect of the treatment with GnRHa in children with genetic short stature and rapidly progressive puberty. Studies presenting adult height data after treatment with GnRH agonists alone are few. Carel et al^[^^1^^]^ treated 31 girls with idiopathic short stature and onset of puberty around the age of 12 for an average of 1.9 years. They reported disappointing results since adult over pretreatment-predicted height increment was 1±2.3 cm (*P*<0.02)^[^^9^^]^. They also reported marked decline in growth velocity during treatment and increased height deficit by 0.4 standard deviation score (SDS) on average in these already short girls. Our results also show that the growth velocity was highest during the first 6 m after beginning of treatment and declined thereafter which is supported by the study of Carel et al ^[^^1^^]^.

 Yanovski et al^[^^10^^]^ conducted a placebo-controlled randomized study in NIH on a heterogeneous population using GnRHa with a mean duration of treatment of 3.5 years. They showed that adult height SDS increased and the difference was about 4.2 cm. They also stated that their treatment was associated with decreased bone mineral density.

 Although the results of these two investigations seem discrepant, but they both indicate that reduced growth rate and reduced bone age progression are two opposite effects of treatment with GnRHa. If the duration of treatment is short, as in the study of Carel et al and the present study, no effect on the final height is seen. Lazar et al^[^^11^^]^ also had a similar observation in which short duration of treatment with GnRHa had little or no clinically significant gain in the adult height. But if the duration increases, as in the study of Yanovski et al, the absence of the progression of bone age combined with the slow growth rate, eventually leads to increased adult height. The mean effect has been estimated to be close to 1 cm of height gain per year of treatment.

 There are also other approaches to increase adolescent growth, namely: Growth hormone alone, growth hormone in combination with GnRHa, sex steroids (testosterone in particular) and aromatase inhibitors. These have their specific indications and studies have been carried out regarding their efficacy and safety^[^^23^^-^^26^^]^.

 The combination of growth hormone and GnRHa is a popular approach for children born small for gestational age or with a diagnosis of idiopathic short stature. Several encouraging studies have shown variable effects but only in a few of them a relevant control group has been included and adult height data should be measured in future studies^[^^22^^, ^^27^^-^^29^^]^.

 Studies addressing the auxological effect of GnRHa in treatment of central precocious puberty have mainly focused on FH outcomes and body weight changes have been ignored to some extent. It is also of note that obesity in childhood is associated with early puberty, and during past two decades, we are witnessing a doubled prevalence of overweight among youth^[^^19^^-^^21^^]^. That is why the effect of GnRHa treatment on body weight is now more important. Carel et al^[^^21^^] ^have shown that BMI increases during treatment with GnRHa, especially in patients with hypothalamic hamartoma and precocious puberty. Feuillan et al^[^^28^^]^ and Boot et al^[^^29^^]^ also showed that GnRHa treatment in central precocious puberty increases the percentage of fat mass and BMI SDS for chronological age. On the other hand, reports of Arrigo et al^[^^26^^]^ and Lebrethon et al^[^^27^^]^ indicate that BMI decreases during these treatments or that the increase in weight is not significantly affected by GnRHa.

 In one recent study performed in Shiraz, Iran, on GnRHa treatment for children with idiopathic central precocious puberty, it was shown that these agents do not cause metabolic syndrome after 3 and 6 months of therapy, and they might only induce hyperlipidemia and central obesity^[^^30^^]^.

 In the present study, it is concluded that BMI is not correlated to the FH or the PAH during treatment with GnRHa in girls with idiopathic short stature and rapidly progressive puberty. We suggest that the result of these therapies is not significantly affected by higher or lower BMIs. Considering many different results in the limited literature available on this issue, further long term studies are required to clearly explain these controversies. We divided our patients to different groups in order to be able to compare them and so was abated the limitation of not having a separate control group.

## Conclusion

We conclude that girls with genetic short stature and rapidly progressive puberty, who are at risk of not attaining their desired adult height by the relatively early onset of puberty, will not benefit receiving a course of one-year treatment with GnRHa. It is also concluded that BMI can increase significantly but there is no significant difference between the final height and final weight among children with lower or higher BMIs. It means that no advantage exists for girls with lower BMI in gaining taller stature or no disadvantage for obese girls in remaining short despite treatment.

## Authors’ Contribution

Z. Karamizadeh: Concept and design, critical revision of the manuscript.

A. Amirhakimi: Acquisition of data, data analysis, manuscript preparation, and critical revision of the manuscript.

Gh. Amirhakimi: Concept and design, critical revision of the manuscript.

All authors approved the final version of the manuscript.
